# Activation of SIRT1 promotes membrane resealing via cortactin

**DOI:** 10.1038/s41598-022-19136-1

**Published:** 2022-09-12

**Authors:** Naotoshi Iwahara, Kuya Azekami, Ryusuke Hosoda, Iyori Nojima, Shin Hisahara, Atsushi Kuno

**Affiliations:** 1 Department of Pharmacology, Sapporo Medical University School of Medicine, Sapporo, Japan; 2 Department of Neurology, Sapporo Medical University School of Medicine, Sapporo, Japan

**Keywords:** Actin, Protein translocation, Pharmacology

## Abstract

Muscular dystrophies are inherited myopathic disorders characterized by progressive muscle weakness. Recently, several gene therapies have been developed; however, the treatment options are still limited. Resveratrol, an activator of SIRT1, ameliorates muscular function in muscular dystrophy patients and dystrophin-deficient *mdx* mice, although its mechanism is still not fully elucidated. Here, we investigated the effects of resveratrol on membrane resealing. We found that resveratrol promoted membrane repair in C2C12 cells via the activation of SIRT1. To elucidate the mechanism by which resveratrol promotes membrane resealing, we focused on the reorganization of the cytoskeleton, which occurs in the early phase of membrane repair. Treatment with resveratrol promoted actin accumulation at the injured site. We also examined the role of cortactin in membrane resealing. Cortactin accumulated at the injury site, and cortactin knockdown suppressed membrane resealing and reorganization of the cytoskeleton. Additionally, SIRT1 deacetylated cortactin and promoted the interaction between cortactin and F-actin, thus possibly enhancing the accumulation of cortactin at the injury site. Finally, we performed a membrane repair assay using single fiber myotubes from control and resveratrol-fed mice, where the oral treatment with resveratrol promoted membrane repair ex vivo. These findings suggest that resveratrol promotes membrane repair via the SIRT1/cortactin axis.

## Introduction

Muscular dystrophies (MDs) are inherited myopathic disorders characterized by progressive muscle weakness and disability^1^. Mutations in a wide range of proteins, including the dystrophin-associated glycoprotein complex that connects the myofiber cytoskeleton to the extracellular matrix, have been indicted in the pathology of MDs. Complete loss of dystrophin function causes Duchenne muscular dystrophy (DMD), the most common and lethal X-linked myopathy, whereas a partial loss results in Becker MD, having a milder phenotype.

Increased muscle injury after high-intensity exercise has been reported in patients with DMD and animal models^2^. Cycles of contraction and relaxation in skeletal muscles induce cellular membrane friction and strain, causing membrane rupture. Plasma membrane disruption is rapidly resealed by several membrane repair mechanisms for cell survival^3^. F-actin promptly accumulates at the membrane disruption sites to reorganize the cytoskeleton, which functions as a base for recruiting other membrane repair molecules^4^. Rho GTPases such as Rho and Cdc42 have been reported to remodel the actin cytoskeleton during membrane repair^5^.

Cortactin (CTTN), a prominent actin-binding protein, plays a vital role in the assembly of branched actin and in maintaining its stability^6^. CTTN promotes the formation and maintenance of lamellipodia–a plate-like extension of cells that plays a crucial role in migration^7^. Its cellular functions have been attributed to Arp2/3 complex activation, which stimulates actin branch nucleation and recruitment of Rho GTPase regulators^7^. However, the role of CTTN in membrane resealing remains unclear.

The NAD^+^-dependent protein deacetylase SIRT1 is one of seven members of the sirtuin family and is a mammalian homolog of yeast SIR2 (silent information regulator 2), the overexpression of which elongates the yeast lifespan^8^. Previously, we showed that resveratrol (3,5,4-trihydroxy-trans-stilbene; Rsv), an activator of SIRT1, decreases muscular and cardiac oxidative damage and improves pathophysiological conditions in animal DMD models^9–13^. We performed a clinical trial to determine the effects of Rsv in 11 patients, including DMD, Becker MD, and Fukuyama MD. We found an improved motor function and an attenuated serum creatine kinase (CK) activity after oral treatment with Rsv^14^. Therefore, SIRT1 activation may counteract the fragility of the membranes associated with muscular dystrophies. We also found that skeletal muscle-specific SIRT1 knockout (SIRT1-mKO) mice are prone to exercise-induced CK leakage and have a mild dystrophic phenotype^15^. Additionally, pharmacological inhibition or knockdown of SIRT1 severely disturbs the dynamic aggregation of membrane vesicles at the injured site in C2C12 myoblasts, indicating that SIRT1 participates in the membrane repair system^15^. However, it is still unclear how SIRT1 regulates membrane repair and whether Rsv promotes membrane repair in skeletal muscles.

In the current study, we hypothesized that SIRT1 activation promotes membrane resealing via the reorganization of the cytoskeleton by regulating CTTN. The rationale for this hypothesis is two-fold. First, we previously reported that SIRT1 promotes cell migration by inducing lamellipodia formation by actin filament^16^. Second, SIRT1 deacetylates CTTN, thus promoting lamellipodia formation through the branching of F-actin^17^. To test this hypothesis, we performed membrane repair assays using C2C12 myoblasts, myotubes, and single fiber myotubes from mice and confirmed that Rsv promoted membrane resealing after membrane injury via SIRT1 and cortactin activation. We also demonstrated that SIRT1 deacetylated CTTN, inducing actin accumulation at the injury site upon membrane injury.

## Results

### SIRT1 activators promote membrane resealing in C2C12 cells

**Figure 1:**
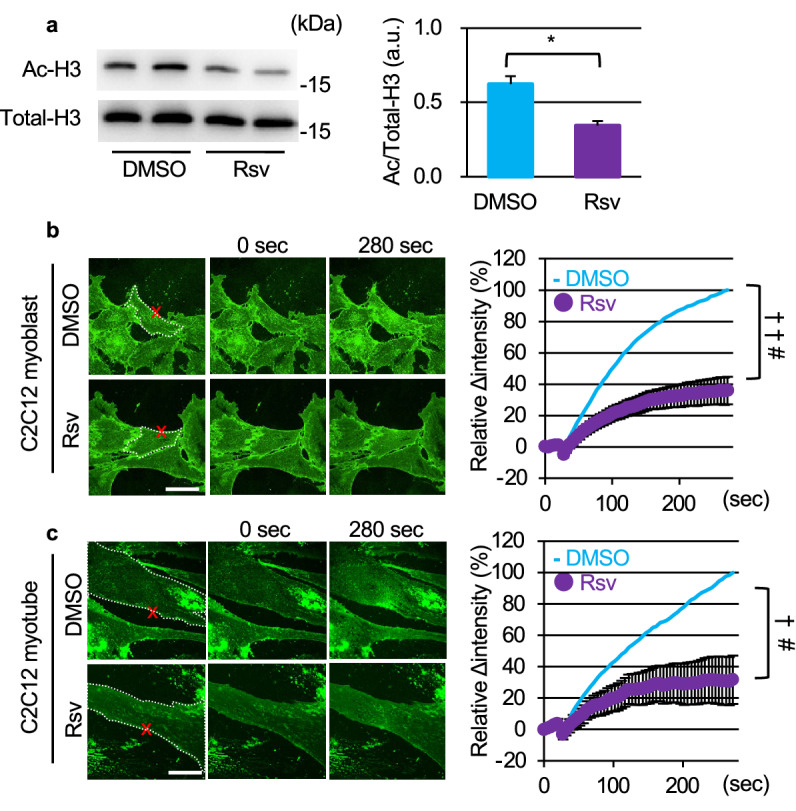
Resveratrol promotes membrane resealing in C2C12 myoblasts and myotubes. (**a**) Immunoblots of acetylated (Ac-; top) and total (bottom) histone H3 in C2C12 cells 12 h after treatment with a vehicle (DMSO) or 50 μM of resveratrol (Rsv; n = 3). (**b**) Plasma membrane repair kinetics upon laser injury measured by membrane-impermeable FM_1-43_ dye influx (green). Representative images before and after the laser injury of DMSO- (top) and Rsv- (bottom) treated C2C12 cells. X-marks (red) indicate laser injury points. Dotted lines (white) indicate cellular shapes and it is the region of FM_1-43_ dye influx measured. Right panel shows the time course of FM_1-43_ dye influx after laser injury in C2C12 cells myoblasts treated with DMSO or Rsv (n = 11 and 20). (**c**) Representative images (left) and time course of FM_1-43_ dye influx (right) before and after laser injury in C2C12 myotubes treated with DMSO (top) or Rsv (bottom) (n = 18). Scale bars of images are 60 μm (**b** and **c**). Data are represented as means ± SD (**a**) or SEM (**b** and **c**). A significant difference was determined by a two-tailed Student’s *t*-test: *P < 0.05. For (**b**) and (**c**), † indicates *P* < 0.05 at the last time point (280 s), and # indicates *P* < 0.05 based on the AUC value (area under the curve).

Initially, we examined whether Rsv, a SIRT1 activator, promotes membrane resealing in C2C12 cells. C2C12 myoblasts were incubated with 50 μM Rsv for 12 h. Rsv induced the deacetylation of histone H3 at lysine 9 (H3K9) in C2C12 myoblasts, causing SIRT1 activation (Fig. 1a). Membrane resealing can be monitored by an influx of the fluorescent dye FM_1-43_ in cells after laser irradiation. Pretreatment with 10 μM of cytochalasin D (CyD), actin polymerization inhibitor, enhanced FM_1-43_ uptake, as previously reported (Fig. S1;^18^). In contrast, when cells were treated with Rsv, FM_1-43_ uptake by cells was significantly limited (Fig. 1b, Video S2) compared with those of control cells (Fig. 1b, Video S1). Similar to C2C12 myoblasts, Rsv also promoted membrane resealing after laser irradiation in C2C12 myotubes (Fig. 1c, Videos S3 and 4). Additionally, 10 mM of nicotinamide mononucleotide (NMN), a SIRT1 activator, promoted membrane resealing in C2C12 myoblasts and myotubes (Fig. S2). These data indicated that SIRT1 activators promote membrane resealing.

### Rsv promotes membrane resealing via SIRT1 function in C2C12 myoblasts

**Figure 2:**
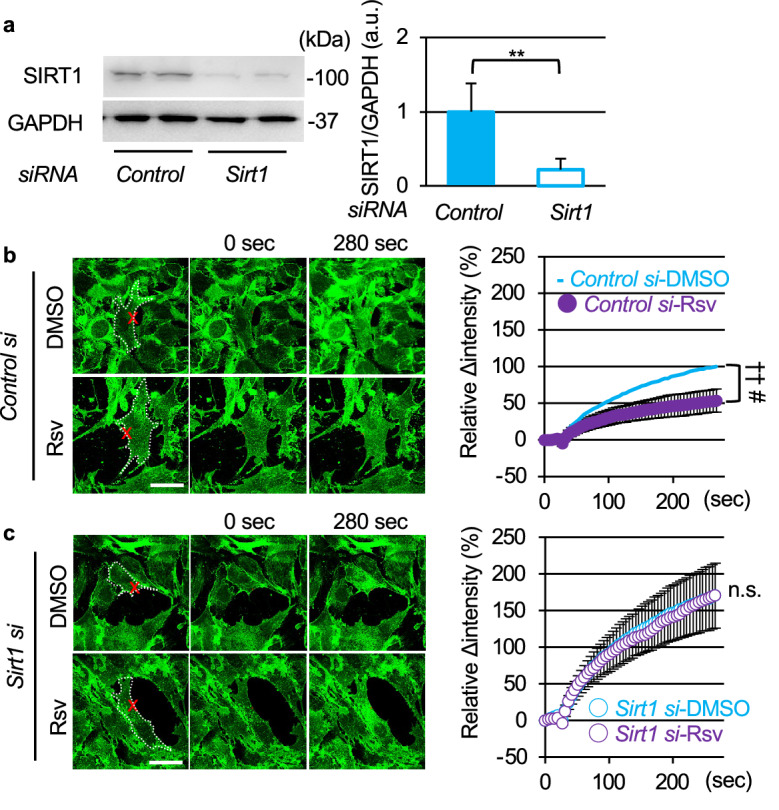
SIRT1 knockdown disturbs promotion of membrane resealing by resveratrol. (**a**) Western blots of C2C12 myoblasts treated with *Control* or *Sirt1 siRNA* for 48 h (n = 6). (**b** and **c**) Left panels show representative images, and right panels show time course of FM_1-43_ dye influx before and after laser injury in C2C12 myoblasts treated with DMSO (top) or Rsv (bottom). C2C12 myoblasts were treated with *Control* (**b**) or *Sirt1 siRNA* (**c**) for 48 h before laser stimulation (*Control si*-DMSO n = 30, *Control si*-Rsv n = 31, *Sirt1 si*-DMSO n = 44, *Sirt1 si*-Rsv n = 45). Data are represented as means ± SD (**a**) or SEM (**b**, **c**). A significant difference was determined by a two-tailed Student’s *t*-test: **P < 0.01. For (**b**) and (**c**), † and †† indicate *P* < 0.05 and 0.01 at the last time point (280 s), respectively. # indicates *P* < 0.05 based on the AUC value. n.s. = not significant.

To assess whether Rsv promotes membrane resealing through SIRT1 activation, we examined the effects of SIRT1 knockdown. C2C12 myoblasts were treated with *control siRNA* or *Sirt1 siRNA* for 48 h. Knockdown of SIRT1 did not change morphology (data not shown) or induce apoptosis^19^. Treatment with *Sirt1 siRNA* reduced SIRT1 protein levels to approximately 20% compared to that in *control siRNA*-treated cells (Fig. 2a). Immunostaining of SIRT1 also showed a diffused reduction of SIRT1 by *Sirt1 siRNA* (Fig. S3). When cells were treated with Rsv, the influx of FM_1-43_ was significantly reduced in *control siRNA*-treated cells (Fig. 2b, Videos S5 and S6). In contrast, *Sirt1 siRNA*-treated cells showed the persistent intracellular entry of FM_1-43_ dye after laser injury, where Rsv failed to promote membrane resealing (Fig. 2c, Videos S7 and S8). This indicated that Rsv promotes membrane resealing through SIRT1 activation.

### SIRT1 is necessary for the reorganization of the cytoskeleton after membrane injury

**Figure 3:**
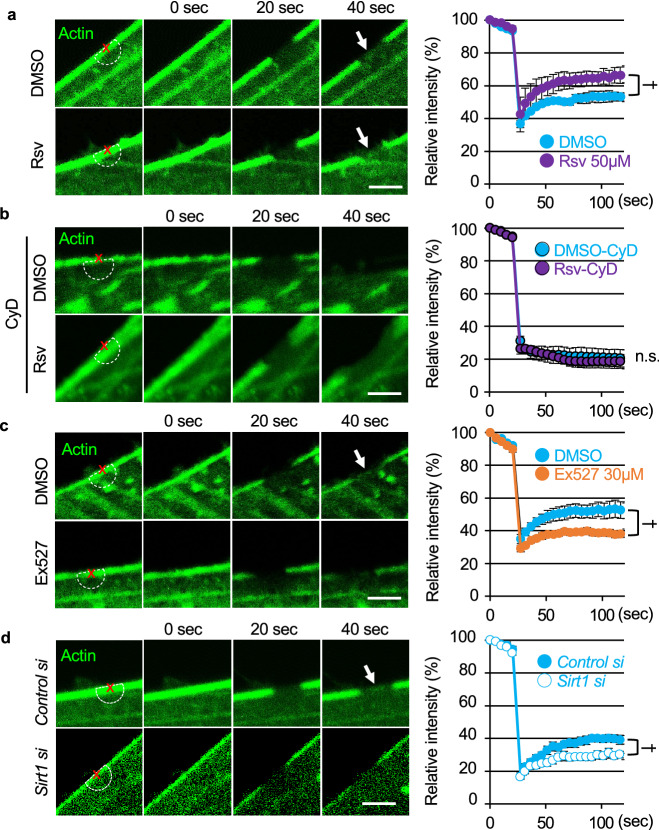
SIRT1 is required for actin accumulation at the damaged site. (**a**–**d**) Accumulation of actin at the laser injured site measured by fluorescence intensity of actin-GFP indicated as semicircle in dotted lines (white; diameter 2 μm). Left panels show representative images, and arrows indicate the accumulation of actin at the injured sites. The right panel shows the time course of the relative intensity of actin-GFP. (**a**) DMSO- (upper) and Rsv- (lower) treated C2C12 cells (n = 10). (**b**) Effect of the depolymerizing condition (cytochalasin D at 10 μM for 30 min) on the promotion of actin accumulation by Rsv (n = 4). (**c**) Effect of a SIRT1 inhibitor, Ex527 (30 μM for 12 h), in C2C12 cells (n = 5). (**d**) Effect of *Sirt1 siRNA* on actin accumulation at the injured site (n = 6). Data are represented as means ± SEM. A significant difference was determined by a two-tailed Student’s *t*-test. † indicates *P* < 0.05 at the last time point (120 s). n.s. = not significant. CyD = cytochalasin D.

Cytoskeleton reorganization occurs during the early phase of membrane resealing^20^. After laser damage, F-actin is enriched at the membrane disruption sites observed in cultured muscle cells and oocytes^21,22^. To determine whether SIRT1 regulates cytoskeleton reorganization, we used C2C12 myoblasts expressing GFP-tagged actin (actin-GFP) and induced membrane damage by laser irradiation. The fluorescence intensity of actin-GFP in the dotted semicircle (Fig. 3) was measured as the actin concentration. Upon laser injury, actin slightly accumulated at the lesion immediately under the sarcolemma (Fig. 3a arrow on the left upper panel, Video S9), as previously reported^21,22^. Interestingly, Rsv-treated cells showed a stronger actin accumulation than control cells (Fig. 3a arrow on the left lower panel, Video S10). These findings indicate that SIRT1 plays a key role in cytoskeletal reorganization following membrane damage. However, when cells were treated with 10 μM of CyD, accumulation of actin was not observed, and the membrane was disarranged regardless of treatment with Rsv (Fig. 3b, Videos S11 and 12). We next examined the effects of SIRT1 specific inhibitor, Ex527, and the knockdown of SIRT1 on actin reorganization. Ex527 at 30 μM or *siRNA* was treated for 12 h or 48 h respectively. In resting *Sirt1 siRNA*-treated C2C12 cells, actin-GFP fluorescence intensity tended to be weaker and cortical actin appeared to be disconnected (Fig. S4). This phenomenon might relate to the role of SIRT1 on formation of stressed fiber as reported by Motonishi et al.^23^. Since SIRT1-mKO mice showed fragility for exercise^15^, SIRT1 may maintain structure of muscular cells by regulating actin, sarcolemma and dystrophin complex. And upon laser injury, EX527 and *Sirt1 siRNA* treatment suppressed actin accumulation at the site of injury (Fig. 3c and d, Movies S13–16).

### Cortactin is required for membrane resealing via accumulation of actin at the injury site

**Figure 4:**
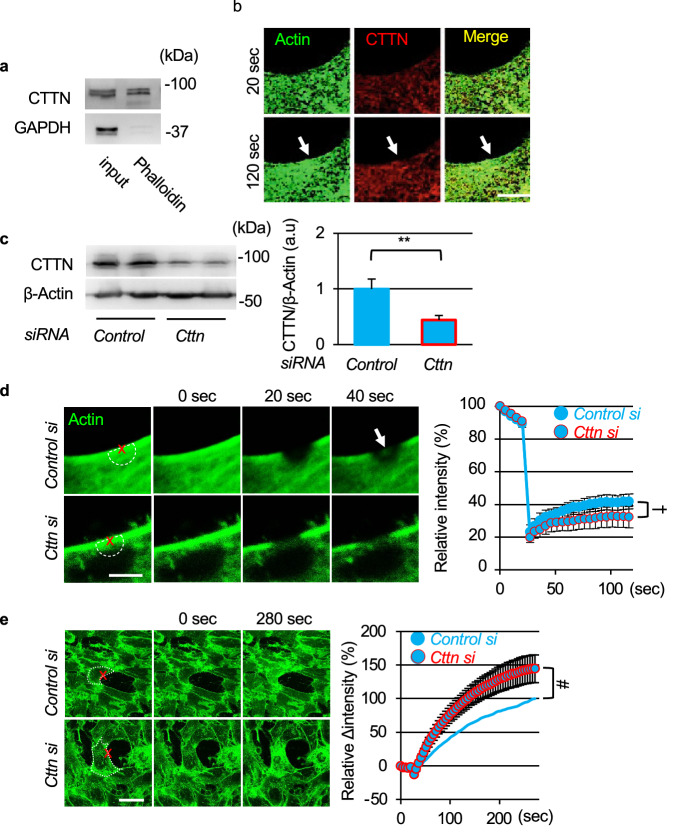
Cortactin is necessary for accumulation of actin on injured site and membrane resealing. (**a**) Western blots for cortactin (CTTN) of C2C12 myoblast lysates and sediment pulled down with Biotin-XX-Phalloidin. (**b**) COS7 cells were transfected with actin-GFP and CTTN-mCherry. Representative images of actin-GFP (green) and CTTN-mCherry (red) immediately after and 4 min after laser injury. Arrows indicate accumulation of actin-GFP and CTTN-mCherry at the injured site. (**c**) Western blots for CTTN of C2C12 myoblasts treated with *Control* or *Cttn siRNA* for 48 h (n = 4). (**d**) *siRNA*-treated C2C12 myoblasts were transfected with actin-GFP (green), and accumulation of actin at the laser injured site was measured by fluorescence intensity of actin-GFP indicated as semicircle in dotted lines (white; diameter 2 μm, n = 6). (**e**) C2C12 myoblasts were treated with *Control* or *Cttn siRNA* for 48 h before laser stimulation (n = 6). Plasma membrane repair kinetics upon laser injury was measured by membrane-impermeable FM_1-43_ dye influx. Data are represented as means ± SD (c) or SEM (**d** and **e**). A significant difference was determined by a two-tailed Student’s *t*-test: **P < 0.01. For (**d**) and (**e**), †indicates *P* < 0.05 at the last time point (120 s), and # indicates *P* < 0.05 based on the AUC value.

Since small membrane protrusions were frequently observed at the injury site and appeared as small lamellipodia (Fig. S5), we focused on the function of CTTN. To confirm the interaction between CTTN and F-actin, we performed a pull-down assay using phalloidin, a mushroom toxin that binds to F-actin. F-actin and its complexes were pulled down with phalloidin-XX-biotin. Immunoblotting showed that the CTTN protein was present in the phalloidin-pulldown fraction (Fig. 4a). Since, we could not get enough florescence intensity of mCherry-tagged CTTN (CTTN-mCherry) in C2C12 cells, we used COS7 cells for evaluating the transport of these proteins upon laser injury. Since COS7 cells formed less cortical actin compared to C2C12 cells (Fig. S6), COS7 cells expressing only CTTN-mCherry did not show CTTN accumulation at the injury site after laser injury. However, COS7 cells co-expressing actin-GFP and CTTN-mCherry showed an accumulation of actin and CTTN at the lesion immediately under the sarcolemma (Fig. 4b arrows, Video S17). This indicates that CTTN plays a role in cytoskeleton reorganization after membrane injury. To assess the function of CTTN in membrane resealing, we used *siRNA* to knockdown CTTN in C2C12 cells. *Cttn siRNA* reduced the CTTN protein levels by approximately 50% (Fig. 4c). Immunostaining of CTTN showed a diffused reduction of CTTN by *Cttn siRNA* (Fig. S3). *Control siRNA*-treated C2C12 myoblasts showed actin accumulation at the injury site (Fig. 4d arrow, Video S18). Treatment with *Cttn siRNA* attenuated actin accumulation (Fig. 4d, Video S19). Additionally, FM_1-43_ uptake upon membrane damage was enhanced in CTTN-knockdown C2C12 cells compared to control cells (Fig. 4e, Videos S20 and S21).

### SIRT1 deacetylates CTTN and promotes CTTN accumulation at the injury site

**Figure 5:**
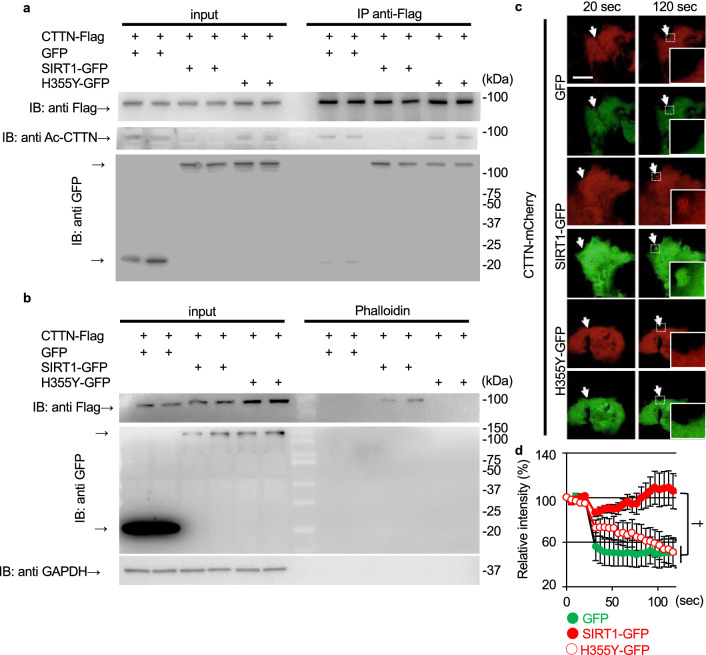
SIRT1 deacetylates cortactin and promotes actin accumulation at the membrane injury site. (**a**) COS7 cells transfected with CTTN-Flag and GFP, SIRT1-GFP, or deacetylase-defective SIRT1 mutant (H355Y)-GFP were lysed for immunoprecipitation. Lysate was immunoprecipitated with anti-Flag mouse IgG and was assessed using anti-Flag antibody (top), anti-acetylated CTTN antibody (Ac-CTTN; middle), and anti-GFP-antibody (bottom). (**b**) Immunoblots for Flag and GFP of lysates and sediments pulled down with Biotin-XX-Phalloidin from COS7 cells expressing CTTN-Flag and GFP, SIRT1-GFP or H355Y-GFP. (**c**) COS7 cells were transfected with CTTN-mCherry and GFP, SIRT1-GFP, or H355Y-GFP. Representative images of GFP (green) and CTTN-mCherry (red) immediately after and 100 s after laser injury. Arrows indicate injured sites. (**d**) The intensity of CTTN-mCherry at the injured site (n = 7). Data are represented as means ± SEM. A significant difference was determined by a one-way ANOVA followed by post hoc comparison with Tukey–Kramer HSD test.: †indicates *P* < 0.05 at last time point (120 s).

Since knockdown of SIRT1 and CTTN resulted in less actin accumulation at the injury site, we assumed that SIRT1 regulates CTTN function required for membrane resealing since SIRT1 deacetylates CTTN and promotes cell migration^17^. To confirm the interaction between SIRT1 and CTTN, COS7 cells expressing GFP-tagged SIRT1 (SIRT1-GFP) and Flag-tagged CTTN (CTTN-Flag) were used. COS7 cells expressing CTTN-Flag and either GFP or SIRT1-GFP were lysed, and further immunoprecipitation was performed using an anti-Flag antibody conjugated with beads. Immunoprecipitation of Flag pulled down CTTN-Flag, and the anti-Flag antibody co-immunoprecipitated SIRT1-GFP but not GFP (Fig. 5a). Additionally, acetylated CTTN-Flag was not detected in COS7 cells co-expressing SIRT1-GFP (Fig. 5a). In COS7 cells, CTTN-Flag was pulled down with phalloidin but was detectable in the phalloidin-pulldown fraction in lysates from cells expressing SIRT1-GFP (Fig. 5b). We used mutant SIRT1 (H355Y), lacking the deacetylation activity of normal SIRT1^24^, to examine whether the deacetylation of CTTN is necessary for binding to F-actin. Lysates from COS7 cells expressing CTTN-Flag and H355Y-GFP were immunoprecipitated with an anti-Flag antibody, and we found that H355Y-GFP co-immunoprecipitated with CTTN-Flag (Fig. 5a). However, CTTN was not deacetylated by co-expression with H355Y-GFP (Fig. 5a) and was not pulled down with phalloidin in cells expressing H355Y-GFP. These results indicate that the deacetylation of CTTN by SIRT1 promotes the binding of CTTN to F-actin.

Next, we examined whether co-expression of SIRT1-GFP induces CTTN accumulation at the injury site (Fig. 5c, arrows). Similar to CTTN-mCherry, SIRT1-GFP did not accumulate at the injury site in COS7 cells expressing only SIRT1-GFP (data not shown). In cells co-expressing CTTN-mCherry and GFP, CTTN-mCherry was not accumulated at the injury site (Fig. 5c upper panels and Fig. 5d, Video S22). When SIRT1-GFP was co-expressed, CTTN-mCherry was accumulated significantly (Fig. 5c middle panels and Fig. 5d, Video S23). In contrast, H355Y-GFP did not promote CTTN accumulation (Fig. 5c lower panels and Fig. 5d, Video S24).

### Rsv induces deacetylation of CTTN and promotes membrane resealing

**Figure 6:**
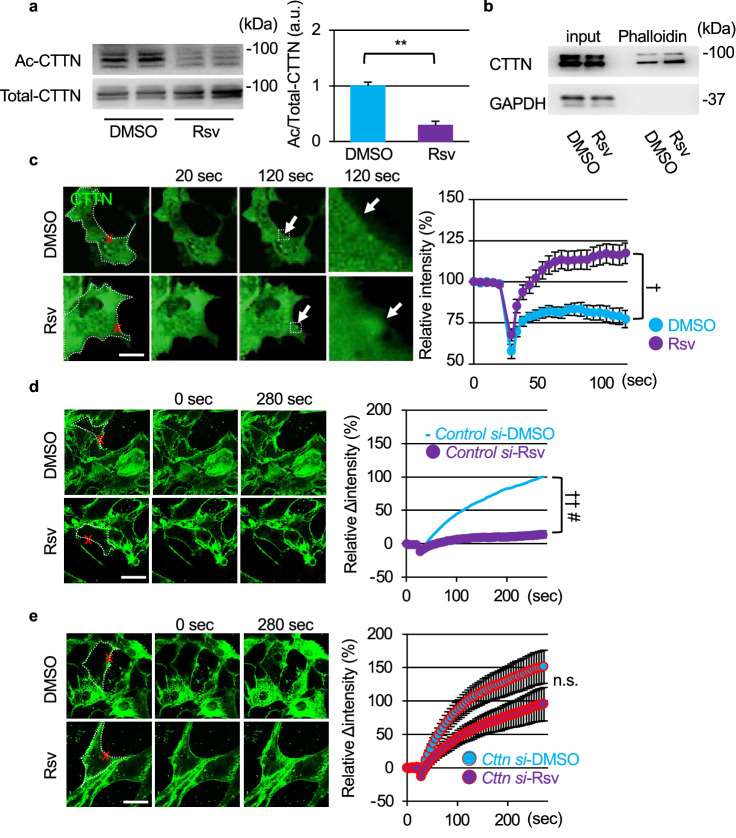
Resveratrol accelerates the accumulation of cortactin at the injured site via deacetylation of cortactin and promotes membrane resealing via cortactin. (**a**) Western blots of acetylated (top) and total (bottom) CTTN in C2C12 myoblasts 12 h after treatment with Rsv and quantification of acetylated CTTN normalize to total-CTTN (n = 3). (**b**) C2C12 myoblasts were treated with DMSO or Rsv and collected. Lysate and sediment pulled down with Biotin-XX-Phalloidin were assessed using anti-CTTN antibody. (**c**) COS7 cells were transfected with CTTN-mCherry. Representative images of CTTN-mCherry (green) immediately after and 100 s after laser injury. Arrows indicate injured sites. The right panel shows the intensity of CTTN-mCherry at the injured site (n = 11). (**d** and **e**) Left panels show representative images, and right panels show the time course of FM_1-43_ dye influx before and after laser injury in DMSO- (top) and Rsv-(bottom) treated C2C12 myoblasts. C2C12 myoblasts were treated with *Control* (D, n = 12) or *Cttn siRNA* (E, n = 12) for 48 h before laser stimulation. Data are represented as means ± SD (**a**) or SEM (**c**, **d**, and **e**). A significant difference was determined by a two-tailed Student’s *t*-test: **P < 0.01. † and †† indicate *P* < 0.05 and 0.01 at the last time point (120 s or 280 s), respectively. # indicates *P* < 0.05 based on the AUC value. n.s. = not significant.

Our data indicate that SIRT1 regulates membrane resealing through the deacetylation of CTTN. To evaluate the effects of Rsv on CTTN, C2C12 myoblasts were treated with 50 μM Rsv, and further immunoblotting was performed for acetylated CTTN. CTTN was deacetylated by Rsv treatment (Fig. 6a). The phalloidin-pulldown fraction from Rsv-treated C2C12 myoblasts contained more CTTN than that from control cells, indicating that Rsv enhanced the binding of CTTN to F-actin (Fig. 6b). In COS7 cells expressing CTTN-mCherry, Rsv treatment induced the accumulation of CTTN at the injury site, while no accumulation was found in control cells (Fig. 6c, Videos S25 and S26). These results indicate that Rsv promotes the deacetylation of CTTN, which enhances the binding of CTTN to F-actin needed for membrane resealing. To evaluate the role of CTTN in the effect of Rsv on membrane resealing, we knocked down CTTN expression in C2C12 cells and monitored membrane resealing by an influx of the fluorescent dye FM_1-43_. Rsv treatment improved membrane resealing in *control siRNA*-treated C2C12 myoblasts (Fig. 6d, Videos S27 and S28). Although treatment with Rsv tended to suppress the entry of FM_1-43_ dye into CTTN-knockdown cells, there was no statistically significant difference compared to vehicle (dimethyl sulfoxide; DMSO)-treated cells (Fig. 6e, Videos S29 and S30).

### Rsv improves membrane resealing of single fiber myotube ex vivo

**Figure 7:**
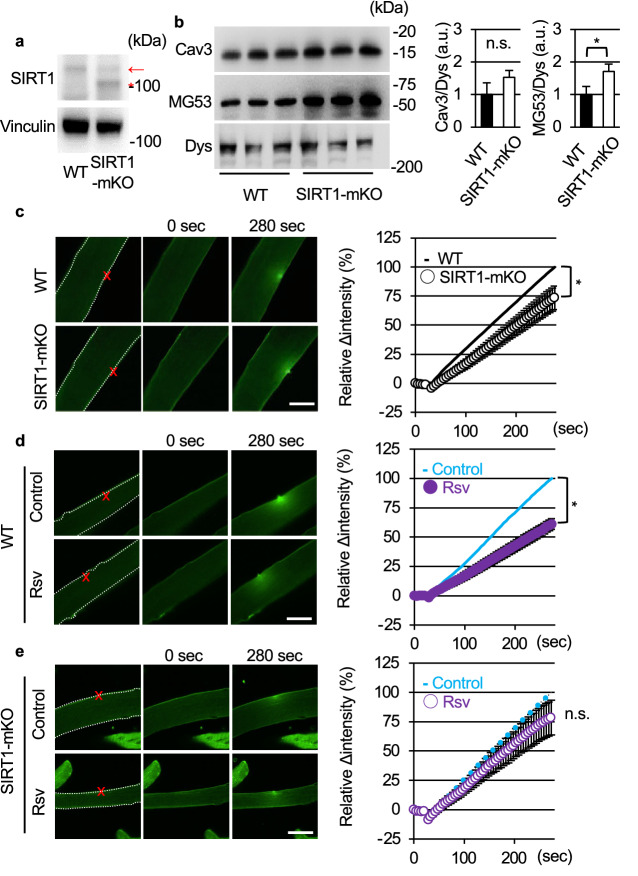
Resveratrol promotes membrane resealing ex vivo*.* (**a**) Immunoblots of SIRT1 (top) and Vinculin (bottom) in tibialis anterior of WT and SIRT1-mKO mice. The band of wild SIRT1 is detected around 110 kDa (red arow), whereas the band of mutant SIRT1, which defects amino acid sequence derived from *Sirt1* gene exon 4, is found at 100 kDa (red asterisk). (**b**) Immunoblots of caveolin 3 (Cav3), MG53 and dystrophin (Dys) in tibialis anterior of WT and SIRT1-mKO mice (n = 3). (**c**) Representative images (left) and time course of FM_1-43_ dye influx (right) before and after laser injury in single fiber myotubes from litters (WT) and SIRT1-mKO mice (15 fibers and 16 fibers from 3 mice respectively). The dotted lines indicate the region of FM_1-43_ dye influx measured. (**c** and **d**) Left panels show representative images, and right panels show time course of FM_1-43_ dye influx before and after laser injury in single fiber myotubes from untreated (control) and Rsv-treated (Rsv) mice. WT (**d**) and SIRT1-mKO (**e**) mice were orally administered with control or 0.4 g Rsv/kg of food ad libitum for 5 days. Eighteen fibers from 3 untreated WT mice and 18 fibers from 3 Rsv-treated WT mice were analyzed in (**d**). Eighteen fibers from 3 untreated SIRT1-mKO mice and 19 fibers from 3 Rsv-treated SIRT1-mKO mice were analyzed in (**e**). Data are represented as means ± SD (**b**) or SEM (**a**, **c** and **d**). A significant difference was determined by a two-tailed Student’s *t*-test: *P < 0.05. For (**c**), (**d**) and (**e**), † indicate *P* < 0.05 at the last time point (280 s). n.s. = not significant.

We previously reported that the muscle of SIRT1-mKO mice was prone to being damaged by exercise^15^, and SIRT1-mKO mice were used to analyze membrane repair function ex vivo. In SIRT1-mKO mice, mutant SIRT1 of about 100 kDa, which lacked amino acid sequence coded by exon 4 of the *Sirt1* gene, was expressed (Fig. 7a). Because SIRT1-mKO mice have a mild dystrophic phenotype^15^, we evaluated protein levels of other membrane repair proteins, caveolin3 (Cav3) and mitsugumin 53 (MG53;^24^). Cav3 tended to be higher in SIRT1-mKO mice tibialis anterior muscle than that in WT mice, and MG53 was significantly higher in SIRT1-mKO mice than WT mice (Fig. 7b).

To confirm the function of SIRT1 and effects of Rsv on membrane resealing ex vivo, we separated single fiber myotubes from the flexor digitorum brevis (FDB) of mice. First, we took FDB single fiber myotubes from SIRT1-mKO mice and their litters (WT), and membrane resealing was monitored, as in the case of C2C12 myotubes. Unexpectedly, SIRT1-mKO mice showed significantly limited influx of FM_1-43_ compared to WT (Fig. 7c). Next, WT mice were fed a control diet or diet including a 0.4 g Rsv/kg for 5 days. FM_1-43_ uptake in single fiber myotubes from Rsv-fed WT mice was significantly limited (Fig. 7d, Video S32) compared to that in control WT mice (Fig. 7d, Video S31). In contrast, Rsv-diet failed to promote membrane resealing in SIRT1-mKO mice (Fig. 7e), indicating that Rsv promoted membrane resealing via SIRT1 ex vivo.

## Discussion

In the current study, we found two new findings. First, SIRT1 activators promote plasma membrane repair via CTTN. Second, CTTN is involved in plasma membrane repair. We showed that Rsv promotes membrane resealing upon injury in C2C12 myoblasts, myotubes, and single myofibers in mice (Figs. 1 and 7). Since the effect of Rsv on membrane resealing was completely disturbed by knockdown of SIRT1, SIRT1 is shown to be mainly involved in the promotion of membrane resealing induced by Rsv (Fig. 2). Additionally, NMN, a SIRT1 activator, promoted membrane repair (Fig. S2). This is the first study to demonstrate the effect of SIRT1 activation on membrane resealing. We have previously reported that Rsv alleviates the phenotype of *mdx* mice, a model of DMD, and improves motor function in patients with MD^9–14^. We also suggested previously that SIRT1 activation by Rsv improves the pathophysiology of MD by decreasing cellular oxidative stress^9,25^ and promoting autophagy^12,13^. In addition to these mechanisms, the promotion of membrane resealing could be a novel mechanism of Rsv treatment in MD.

We previously reported that SIRT1 inhibition severely disturbed the dynamic aggregation of membrane vesicles at the injury site in C2C12 myoblasts^15^. However, the underlying mechanism has not yet been elucidated. Rsv accelerated actin accumulation at the injury site, and the knockdown of either SIRT1 or CTTN suppressed the formation of actin (Figs. 3d and 4d). SIRT1 and CTTN were also seen to be co-localized at the injury site (Figs. 4b and 5c). We showed that overexpression of SIRT1 and Rsv induced the deacetylation of CTTN and promoted its binding to F-actin (Figs. 5 and 6). These findings suggest that SIRT1 promotes actin accumulation at the injury site via CTTN deacetylation to repair the membrane. The repeated domain in CTTN, which binds to actin^7^, is acetylated by p300, leading to a reduction in its binding affinity to actin^17^. This domain is considered to be a deacetylation site for SIRT1^17^. Similar to HDAC6, another deacetylase^26^, SIRT1 may regulate the affinity between CTTN and F-actin by deacetylation of the repeat domain of CTTN.

This is the first study to show that CTTN participates in membrane resealing. Because CTTN is known to promote cell migration and actin reorganization with members of Rho GTPase via Arp2/3^27^, which plays important roles in membrane resealing^5,28,29^, actin reorganization via Arp2/3 may underlie the mechanism by which CTTN promotes membrane sealing. Actin-GFP accumulated at the injury site upon membrane disruption in C2C12 myoblasts (Fig. 3a); however, this response was not observed in COS7 cells (data not shown). Therefore, COS7 cells needed an inducer, such as the co-expression of CTTN (Fig. 4b). CTTN-Flag was rarely pulled down by phalloidin in COS7 cells (Fig. 5b). CTTN accumulated at the injury sites in cells co-expressing SIRT1 (Fig. 5c) or treated with Rsv (Fig. 6c) but not in cells co-expressing GFP (Fig. 5c) or treated with DMSO as a vehicle (Fig. 6c). These data indicate that the function of CTTN in membrane resealing in COS7 cells might be weaker than that in C2C12 myoblasts.

Knockdown of SIRT1 and CTTN worsened membrane repair in C2C12 myoblasts, and knockdown of SIRT1 completely abolished the effects of Rsv (Fig. 2c). However, the knockdown of CTTN partially nullified the effects of Rsv (Fig. 6e). SIRT1 might also possibly have other targets that regulate membrane resealing. SIRT1 promotes autophagy by deacetylating autophagy-related molecules, such as Atg5, Atg7, and Atg8^30^. Additionally, we previously reported that Rsv induced the expression of autophagic molecules in *mdx* mice^13^. Moreover, few reports have demonstrated a relationship between autophagy and membrane repair^31,32^. Therefore, autophagic molecules could be another SIRT1 target to promote membrane resealing.

We previously reported that the muscle of SIRT1-mKO mice was prone to being damaged by exercise, and pharmacological inhibition of SIRT1 or SIRT1-knockdown severely disturbed membrane resealing after laser irradiation in C2C12 myoblast without change of membrane repair mRNA expression levels^15^. In this study, muscle fibers from SIRT1-mKO mice, however, showed significant promotion of membrane resealing ex vivo, the change was associated with upregulation of MG53 protein (Figs. 7b). Because, such a compensatory upregulation of MG53 was reported in *mdx* mice^33^, there is a possibility that knockout of SIRT1 resulted in increased fragility of muscle fibers and upregulated other membrane repair proteins to promote membrane repair in SIRT1-mKO mice (Fig. S7). These findings suggest that SIRT1 is not essential for plasma membrane repair. Generation of an inducible and skeletal muscle-specific knockout model may clarify this point.

Finally, we showed that myofiber influx of FM_1-43_ from Rsv-treated mice was lower than that from control mice (Fig. 7d), and Rsv failed to promote membrane resealing on myofiber from SIRT1-mKO mice (Fig. 7e). These results indicates that Rsv improved membrane resealing through SIRT1 ex vivo as same as in vitro assay using C2C12 cells (Fig. S7).

Additionally, since there is a limitation that membrane repair assay using myotubes ex vivo is not physiological condition, further development of more physiological methods for analyzing membrane repair function in vivo is needed. Upon membrane damage, overt membrane tears can be observed at injury sites in vitro and ex vivo; however, there is no particular evidence showing membrane tears in injured muscle after exercise^3^. Since membrane resealing is a very rapid phenomenon, it might be difficult to observe in in vivo. However, several evidences support the tear theory. Dysferlin and MG53 knockout mice showed membrane fragility and impaired repair of membrane tears in vitro^34,35^. Recently, mechanisms for slower repairing of skeletal muscle, such as muscle regeneration^36,37^ and nuclear migration^38^, have been the focus of attention. The proliferation of satellite cells and assembly of myonuclei around injury sites have been observed after muscular damage in vivo^37,38^. Further studies are required to clarify the effects of SIRT1 and Rsv on repairing skeletal muscle damage in vivo.

## Methods

### Animals

All in vivo experiments were conducted in strict accordance with the Guide for the Care and Use of Laboratory Animals (Institute of Laboratory Animal Resources, 1996) and were approved by the Animal Care and Use Committee of Sapporo Medical University (17-035_20-07). The animals were housed in a conventional state under adequate temperature (24 ± 2 °C) and relative humidity (50 ± 5%) with a 12/12 h reversed light/dark cycle with access to food and water ad libitum. Skeletal muscle-specific SIRT1 knockout mice were generated by crossing floxed SIRT1 mice (SIRT1^flox/flox^, strain name B6;129-Sirt1tm1Ygu/J) with human α-skeletal muscle actin promoter driven Cre mice (ACTA1-Cre79Jme/J), both obtained from the Jackson Laboratory. Genotypes were confirmed by PCR using primers for Cre (forward: 5’-CGAATAACTACCTGTTTTGCCGGGT-3’, reverse: 5’-TCGCCATCTTCCAGCAGGCGCACCA-3’) and for SIRT1 flox alleles^39^. Mice from 8 months of age were orally administered with control or 0.4 g Rsv/kg of food ad libitum for 5 days. The FDB was removed after deep anesthesia by intraperitoneal injection of xylazine (10 mg/kg) and pentobarbital sodium (50 mg/kg). The mice were then sacrificed by decapitation, conducted only by well-trained persons. Single fibers were isolated from FDB bundles using collagenase digestion. FDB muscles were incubated at 37 °C in Dulbecco’s modified Eagle’s medium (DMEM) containing 0.2‒0.4% (w/v) collagenase type I (Worthington, Lakewood, NJ) and 10% fetal bovine serum (FBS; MP Biomedicals, Aurora, USA) for 1‒2 h. Fibers were separated by gentle trituration in 5 ml of collagenase-free DMEM containing 10% FBS. A medium containing concentrated fiber (200 μl) was placed on a collagen-precoated 11 mm glass dish (11-0612, Iwaki, Japan, Shizuoka). The fibers were then allowed 30 min for attachment. The reporting in the manuscript followed the recommendations in the ARRIVE guidelines.

### Cell culture

C2C12 and COS7 cells were cultured in DMEM with high glucose (Nacalai Tesque, Kyoto, Japan) containing 10% FBS in an incubator with 5% CO_2_ at 37 °C. Twenty-four hours after passage, cells were treated with a vehicle, 30 μM Rsv (FUJIFILM Wako Pure Chemicals, Osaka, Japan), 10 mM NMN (FUJIFILM Wako Pure Chemicals), 1 μM CyD (FERMENTEK, Jerusalem, Israel), or 30 μM Ex527 (Tocris Bioscience, Ellisville, USA) and incubated for 30 min (CyD) or 12 h (Rsv, NMN and Ex527). RNAi-mediated knockdown was performed by transfection of *Sirt1 siRNA* (Sigma-Aldrich, Massachusetts, USA; Mm_Sirt1_5675, 30 nM), *Cttn siRNA* (Sigma-Aldrich; Mm01_00099413, 30 nM), and *control siRNA* (Sigma-Aldrich; Mission_SIC-001, 30 nM) using Lipofectamine RNAiMAX Transfection Reagent (Thermo Fisher Scientific, Massachusetts, USA) according to the manufacturer’s instructions. The experiments were performed 48 h after transfection. CellLight Actin-GFP, BacMam 2.0, (Thermo Fisher Science) was used to overexpress Actin-GFP according to the manufacturer’s instructions. COS7 cells were transfected with the following plasmids using Lipofectamine 3000 Transfection Reagent (Thermo Fisher Scientific) according to the manufacturer’s instructions: pEGFP-N1 (BD Bioscience, New Jersey, USA), SIRT1-GFP, H355Y-GFP^22^, and Cortactin-mCherry (Plasmid #27676, Addgene, Massachusetts, USA). C2C12 cells were cultured in DMEM with low glucose (FUJIFILM Wako Pure Chemicals) containing 2% horse serum (Thermo Fisher Scientific) for 1 week for myotube differentiation.

### Immunoblot analysis

Mammalian Cell Lysis buffer (Sigma Aldrich) was used for C2C12 cells and COS7 cells. Immunoblot analysis were prepared as previously described^15^. The following antibodies were used: anti-Histone H3 (1:10,000 dilution; ab1791, Abcam, Cambridge, UK), anti-Histone H3 acetyl K9 (1:2,000 dilution; ab4441, Abcam), anti-SIRT1 (1:1000 dilution; ab110304, Abcam), anti-CTTN (1:200 dilution, H-191, Santa Cruz Biotech, Texas, USA), anti-acetylated CTTN (1:50 dilution, 09-881, Millipore, Massachusetts, USA), anti-GFP (1:2,000 dilution, ab13970, Abcam), Anti-DYKDDDDK tag (Flag; 1:10,000 dilution, FUJIFILM Wako Pure Chemicals), anti-GAPDH (1:2000 dilution; MAB374, Sigma Aldrich), anti-β-Actin (1:10,000 dilution; 281-98721, FUJIFILM Wako Pure Chemicals) and anti-Vinculin antibody (1:10,000 dilution; V9131, Sigma-Aldrich). Following antibody was used for immunoprecipitation; anti-Flag antibody-conjugated beads (50 μl for each sample, FUJIFILM Wako Pure Chemicals). The immunoprecipitates were washed three times with TBST and analyzed by immunoblotting. For phalloidin pull-down, the lysate was incubated overnight at 4 °C with 5 units of Biotin-XX Phalloidin (B7474, Thermo Fisher Scientific), then incubated with 500 µg of Dynabeads M-280 Streptavidin (11205D, Thermo Fisher Scientific) for 1 h and washed three times with TBST. The pull-downs were analyzed by immunoblotting.

### Immunostaining

For immunostaining, cells were fixed in 4% PFA for 15 min, then blocked with 0.3% Triton X-100, 3% bovine serum albumin and 3% goat serum in phosphate buffered saline (PBS). Sections were incubated with antibodies against SIRT1 (1:1,000 dilution) or CTTN (1:1,000; ab81208, Abcam) overnight at 4 °C. Cells were then probed with secondary antibodies of an anti-mouse IgG antibody conjugated with Alexa Fluor 594 (1:2,000 dilution; Thermo Fisher Science) or an anti-rabbit IgG antibody conjugated with Alexa Fluor 594 (1:2,000 dilution; Thermo Fisher Science). Cells were also stained with Hoechst33342 (Dojindo, Kumamoto, Japan). They were observed by a fluorescence microscope (BZ-9000, KEYENCE, Osaka, Japan).

### Laser damage

For the membrane repair assay, cells with an angular shape were selected, and cells with an obviously high FM_1-43_ uptake compared to surrounding cells were excluded. Stimulation sites were chosen near the center of concave curved edges that were not adherent to other cells. For the observation of actin-GFP dynamics, cells with angular shape, well formation of cortical actin, and sufficient fluorescence intensity were selected. Immediately before the assay, the medium was changed to Live Cell Imaging Solution (Thermo Fisher Scientific) containing 10 µM FM_1-43_ dye (Thermo Fisher Scientific) at 37 °C. Cell membrane damage was induced using a Nikon A1 laser scanning confocal microscope equipped with a plan Apo 100 × oil immersion objective lens (NA 1.4). For laser injury, a 1 µm × 1 µm area was irradiated by a 405 nm laser at 100% power using the photo-activation mode. Each irradiation time was 395.92 ms, and was repeated 15 times (total 20 s). Images were captured using a 488 nm laser. Images were acquired for 20 s every 5 s before the injury, immediately after every injury or after all injuries, and 4 min every 5 s following injury. For each image, the fluorescence intensity of the whole cell was measured using the Nikon NIS Elements v4.1 software. Data are presented as the change in fluorescence intensity relative to the value at 0 s (ΔF/F0) for FM_1-43,_ and fluorescence intensity relative to the value at 0 s for GFP and mCherry. Data from at least four cells were compared in one experiment and were confirmed in three independent experiments.

### Statistical analyses

All data are expressed as mean ± SEM or SD. Differences between groups were analyzed using Student’s *t*-test or one-way ANOVA followed by post hoc comparison with the Tukey–Kramer HSD test. For all tests, statistical significance was set at *P* < 0.05. JMP15 (SAS Institute Inc., Cary, North Carolina, USA) was used for the data analysis.

## Supplementary Information

Supplementary Information 1.

Supplementary Information 2.

Supplementary Video 1.

Supplementary Video 2.

Supplementary Video 3.

Supplementary Video 4.

Supplementary Video 5.

Supplementary Video 6.

Supplementary Video 7.

Supplementary Video 8.

Supplementary Video 9.

Supplementary Video 10.

Supplementary Video 11.

Supplementary Video 12.

Supplementary Video 13.

Supplementary Video 14.

Supplementary Video 15.

Supplementary Video 16.

Supplementary Video 17.

Supplementary Video 18.

Supplementary Video 19.

Supplementary Video 20.

Supplementary Video 21.

Supplementary Video 22.

Supplementary Video 23.

Supplementary Video 24.

Supplementary Video 25.

Supplementary Video 26.

Supplementary Video 27.

Supplementary Video 28.

Supplementary Video 29.

Supplementary Video 30.

Supplementary Video 31.

Supplementary Video 32.

Supplementary Video 33.

Supplementary Video 34.

Supplementary Video 35.

Supplementary Video 36.

Supplementary Video 37.

## Data Availability

All data are contained within this article.
