# Supplementary material for: Activation of SIRT1 promotes membrane resealing via cortactin
Source: Sci Rep. 2022 Sep 12;12:15328. doi: 10.1038/s41598-022-19136-1 (PMC9468153; doi:10.1038/s41598-022-19136-1)

Figure 1a

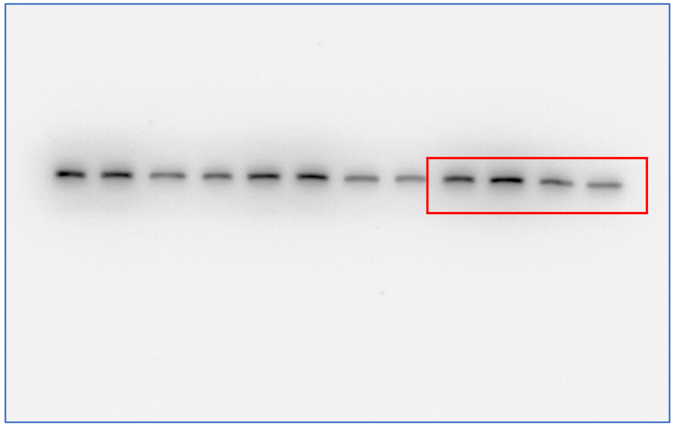

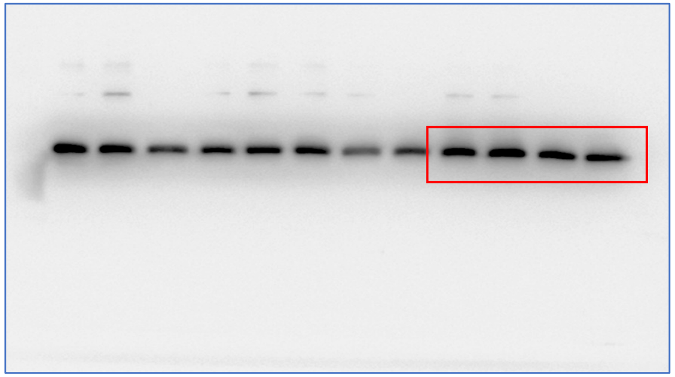

Figure 2a

IB: SIRT1

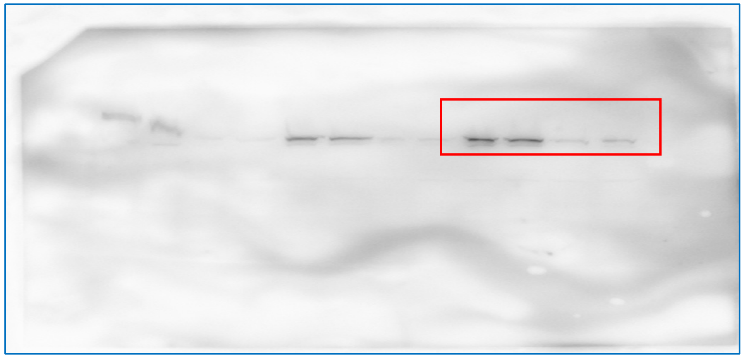

IB: GAPDH

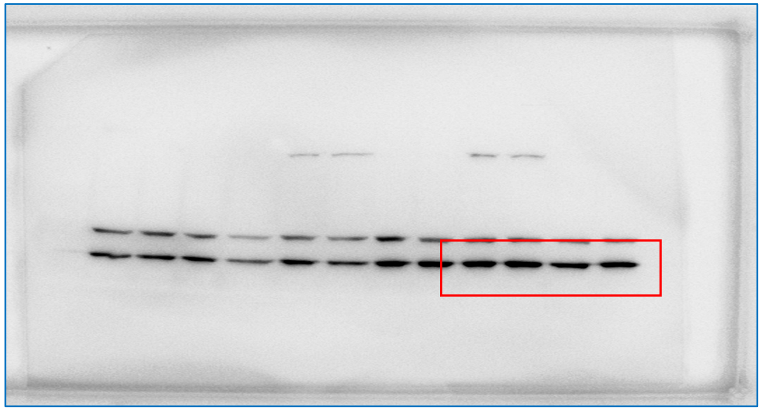

Figure 4a

IB: CTTN

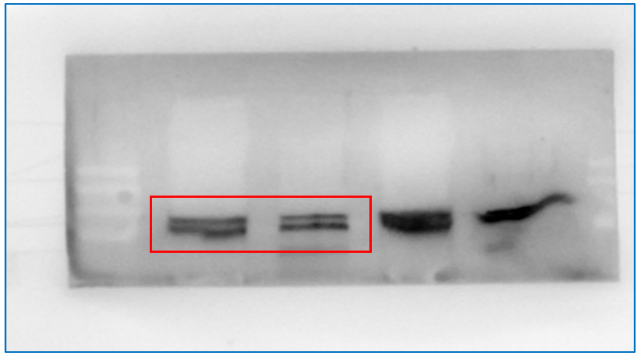

IB: GAPDH

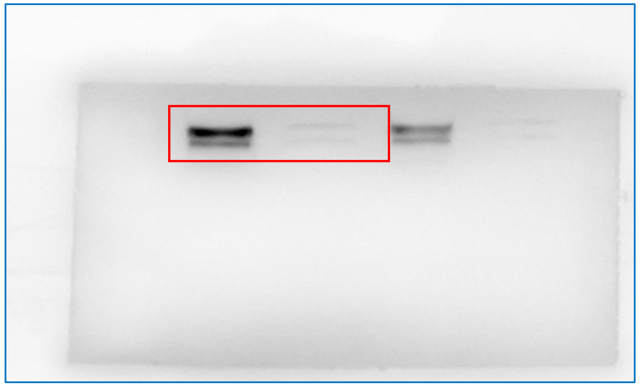

Figure 4c

IB: CTTN

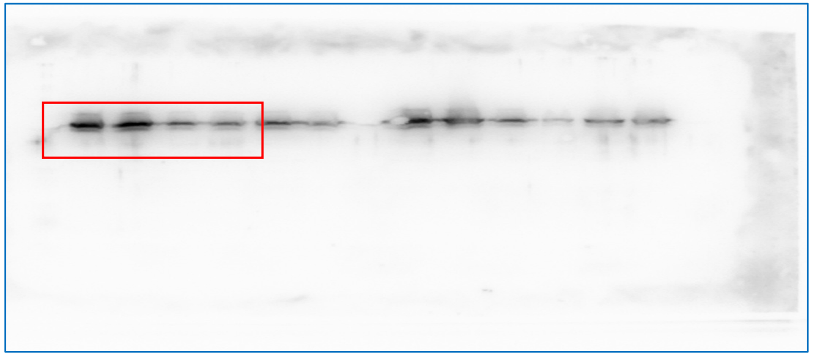

IB:β-Actin

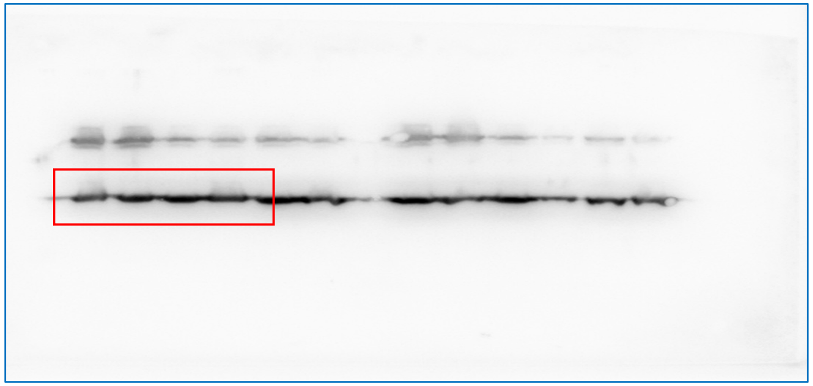

Figure 5a

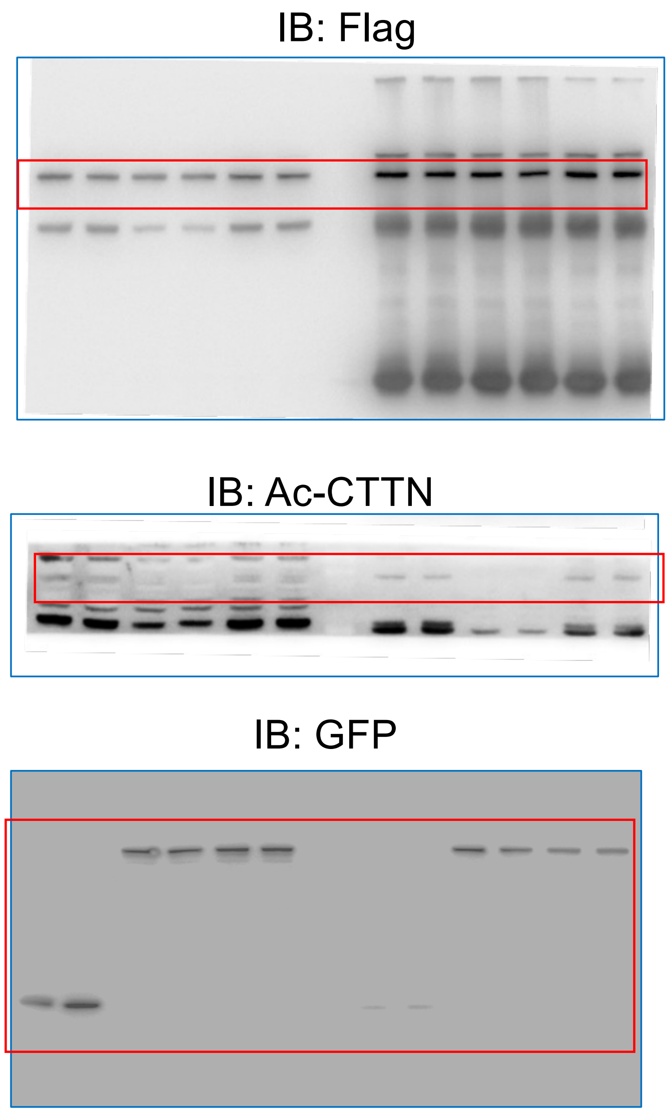

Figure 5b

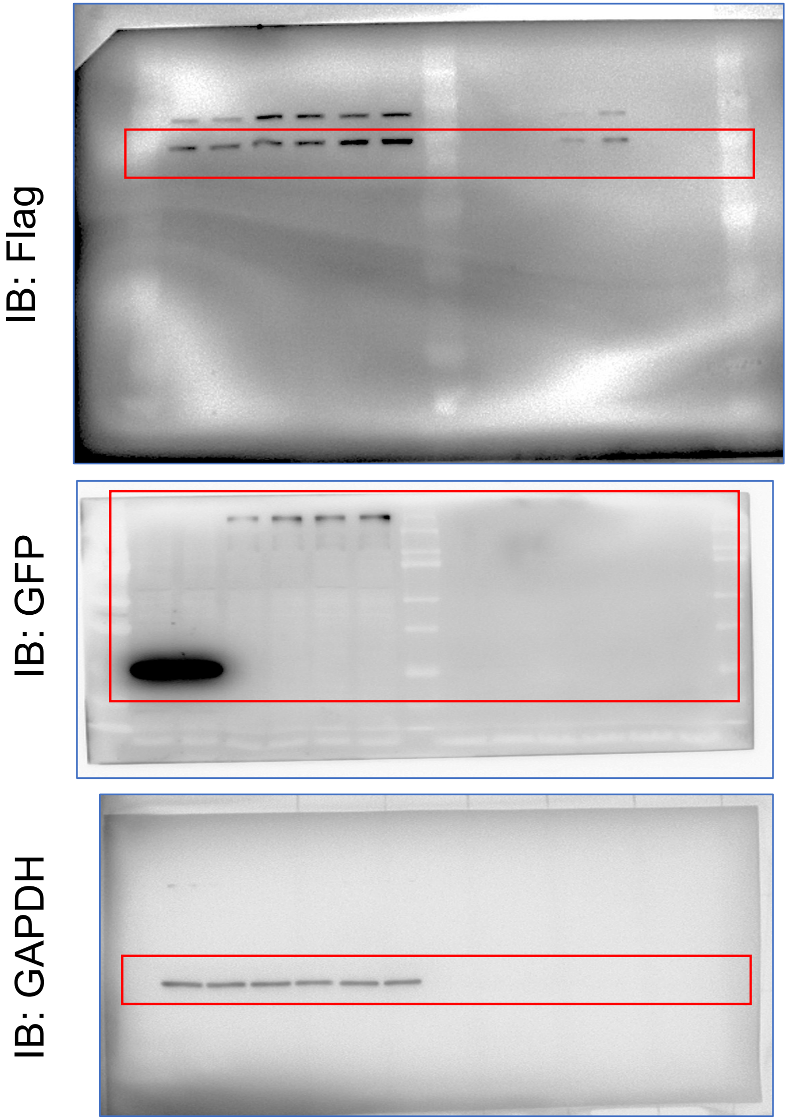

Figure 6a

IB: Ac-CTTN

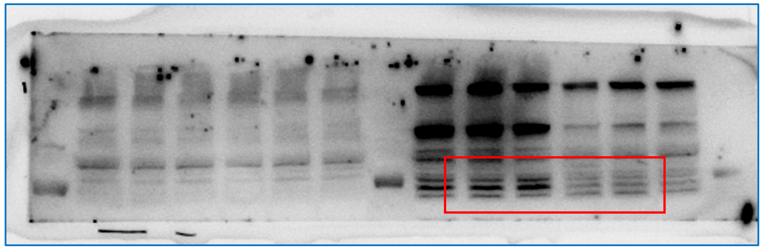

IB: Total-CTTN

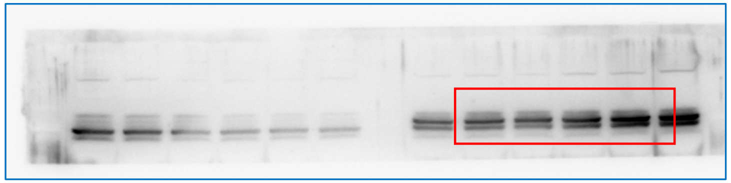

Figure 6b

IB: CTTN

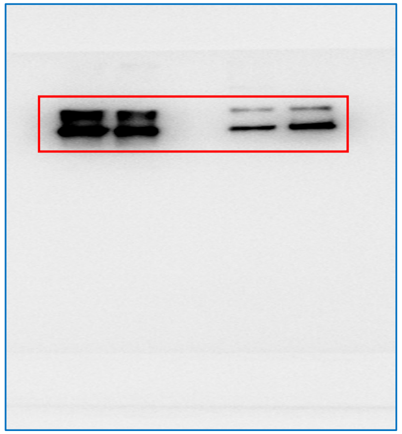

IB: GAPDH

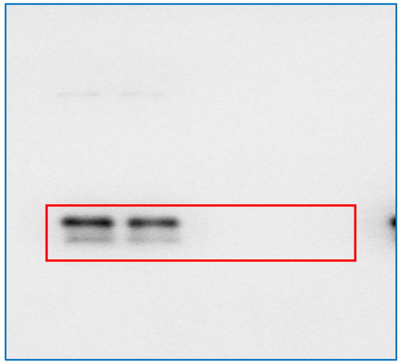

Figure 7a

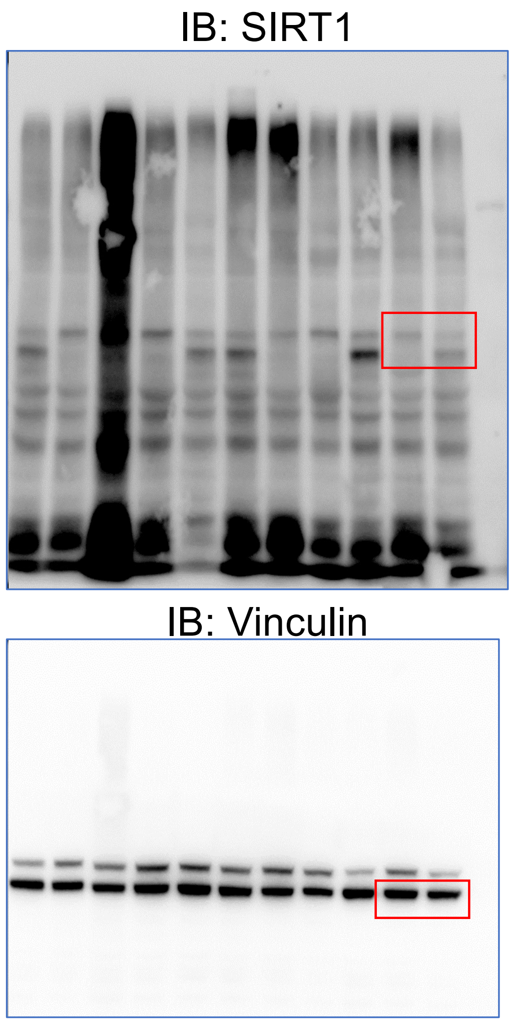

Figure 7b

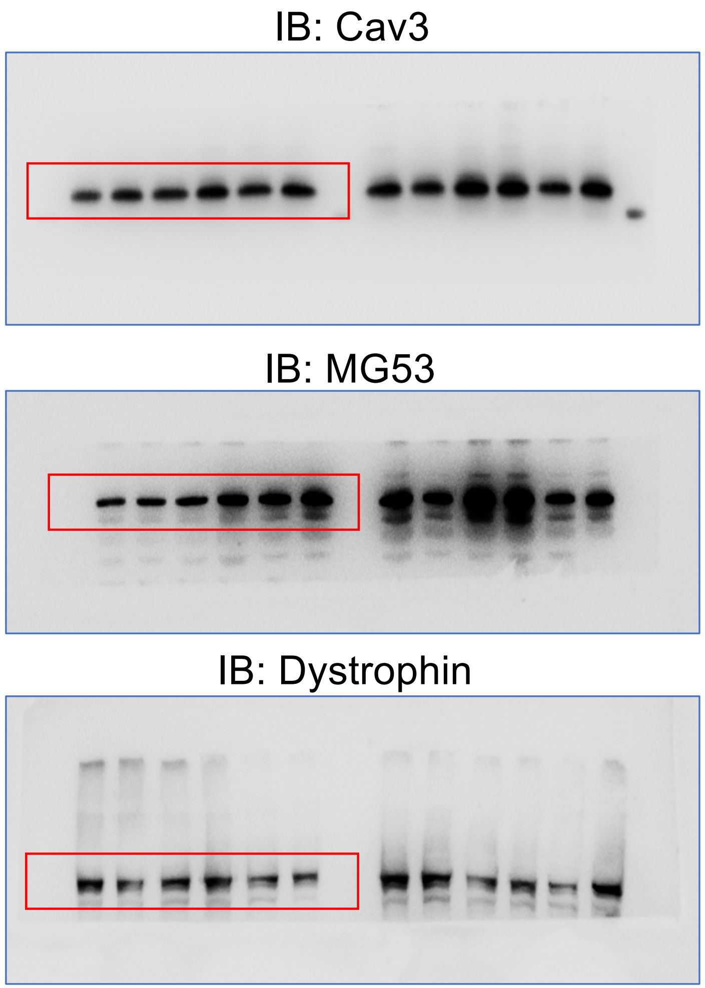

Supplemental Figure 2a

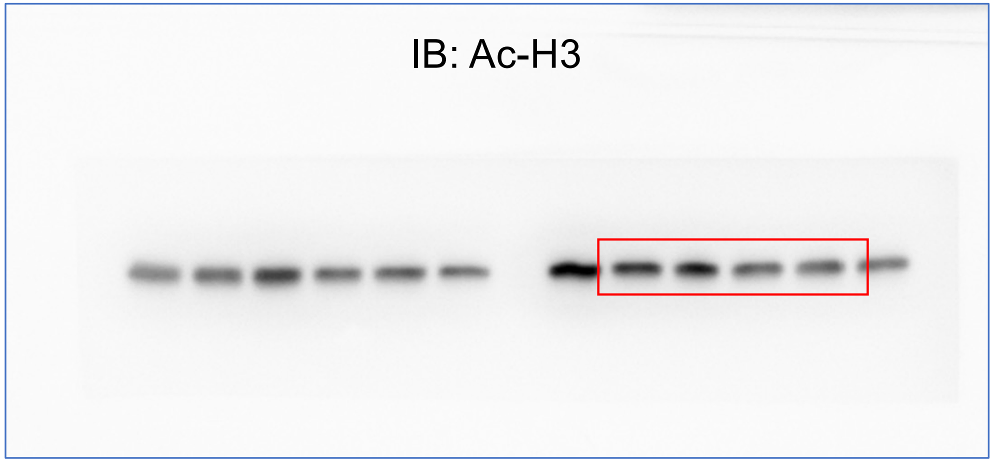

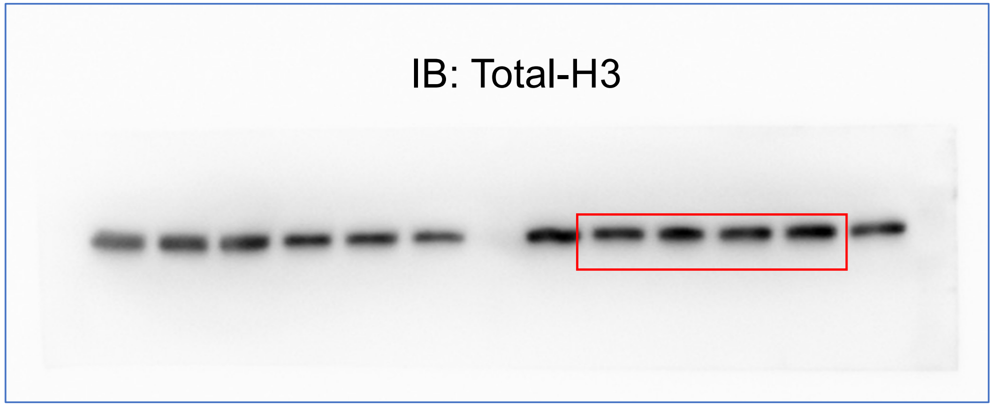

Supplement: Supplementary file 1 — Supplementary Information 1. [file 41598_2022_19136_MOESM1_ESM.pdf]
