# Supplementary material for: Activation of SIRT1 promotes membrane resealing via cortactin
Source: Sci Rep. 2022 Sep 12;12:15328. doi: 10.1038/s41598-022-19136-1 (PMC9468153; doi:10.1038/s41598-022-19136-1)
Supplement: Supplementary file 2 — Supplementary Information 2. [file 41598_2022_19136_MOESM2_ESM.pdf]

Title

Activation of SIRT1 promotes membrane resealing via cortactin.

Authors

Naotoshi Iwahara[1,2], Kuya Azekami[1], Ryusuke Hosoda[1], Iyori Nojima[1], Shin Hisahara[2] and Atsushi Kuno[1*]

[1] Department of Pharmacology, Sapporo Medical University School of Medicine, Sapporo, Japan.
[2] Department of Neurology, Sapporo Medical University School of Medicine, Sapporo, Japan.

[*]Corresponding author
E-mail: kuno@sapmed.ac.jp

Figure S1. Cytochalasin D suppressed membrane resealing in C2C12 myoblasts.

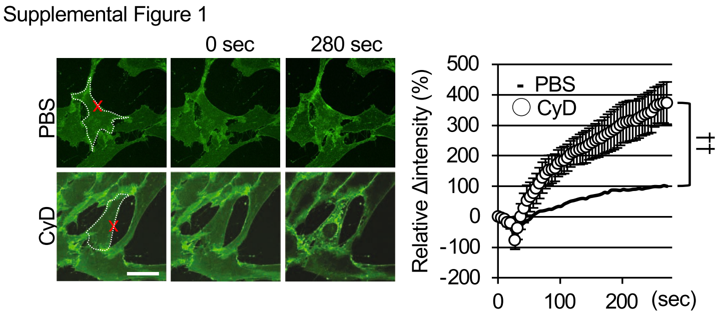

Plasma membrane repair kinetics upon laser injury were measured by membrane-impermeable $FM_{1\text{-}43}$ dye influx (green). Representative images before and after laser injury of PBS- (top) and 10 μM of cytochalasin D (CyD)- (bottom) treated C2C12 cells. X-marks (red) indicate laser injury points, and dotted lines (white) indicate cellular shapes. The right panel shows the time course of $FM_{1\text{-}43}$ dye influx after laser injury in C2C12 myoblasts treated with PBS or CyD (n=4). A significant difference was determined by a two-tailed Student's *t*-test: †† indicate $P<0.01$ at the last time point (280 sec)

Figure S2. Nicotinamide mononucleotide promotes membrane resealing in C2C12 myoblasts and myotubes.

Supplemental Figure 2

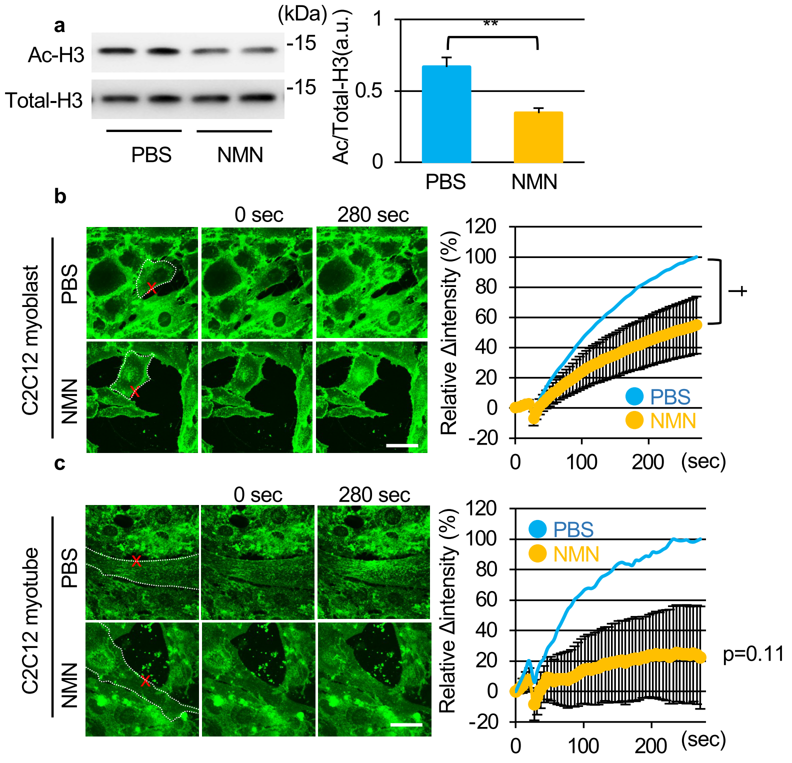

(a) Immunoblots of acetylated (top) and total (bottom) histone H3 in C2C12 cells 12 h after treatment with 10 mM of nicotinamide mononucleotide (NMN; n=3). (b) Plasma membrane repair kinetics upon laser injury measured by membrane-impermeable FM_{1-43} dye influx (green). Representative images before and after laser injury of PBS- (top) and NMN- (bottom) treated C2C12 cells. X-marks (red) indicate laser injury points, and dotted lines (white) indicate cellular shapes. The left panel shows the time course of FM_{1-43} dye influx after laser injury in C2C12 myoblasts treated with PBS or NMN (n=21 and 28, respectively). (c) Representative images (left) and time course of FM_{1-43} dye influx (right) before and after laser injury in C2C12 myotubes treated with PBS (top) or NMN (bottom) (n=24). Scale bars of images are 60 μm (b and c). Data are represented as means ± SD (a) or SEM (b and c). A significant difference was determined by a two-tailed Student's *t*-test: ***P*<0.001. †indicates *P*<0.05 at last time point (280 sec)

Figure S3. Knockdown of SIRT1 and CTTN in C2C12 cells.

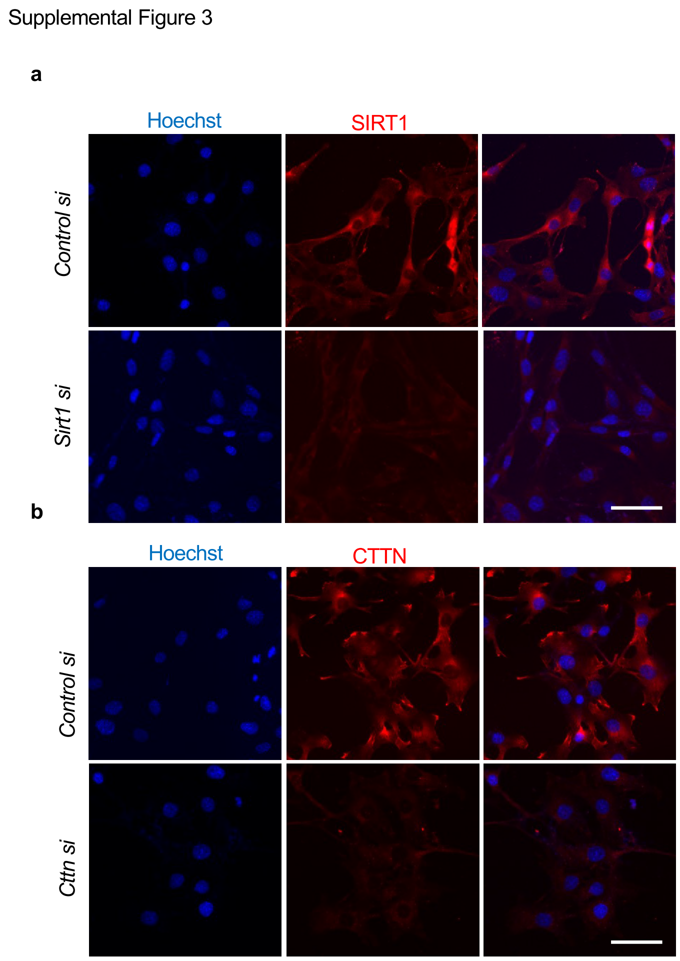

Immunostaining of SIRT1 (a) and CTTN (b) in C2C12 cells 48h after *control, Sirt1* and *Cttn siRNA* treatment. Nuclei were stained with Hoechst33342 (blue). Scale bars of images are 50 μm.

Figure S4. Knockdown of SIRT1 results in disconnected cortical actin.

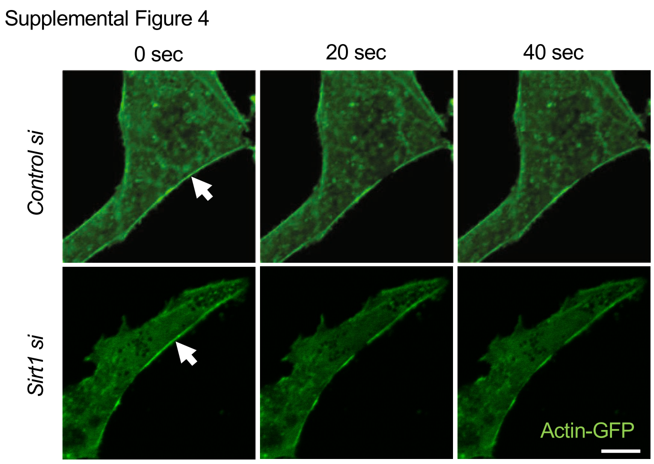

C2C12 cells expressing actin-GFP were treated with *control* or *Sirt1 siRNA*. The plasma membrane of the cells were injured by laser. Cortical actin appeared to be disconnected in *Sirt1 siRNA* treated cell. Scale bar of the image is 5 μm.

Figure S5. Small membrane protrusion appears after actin accumulation.

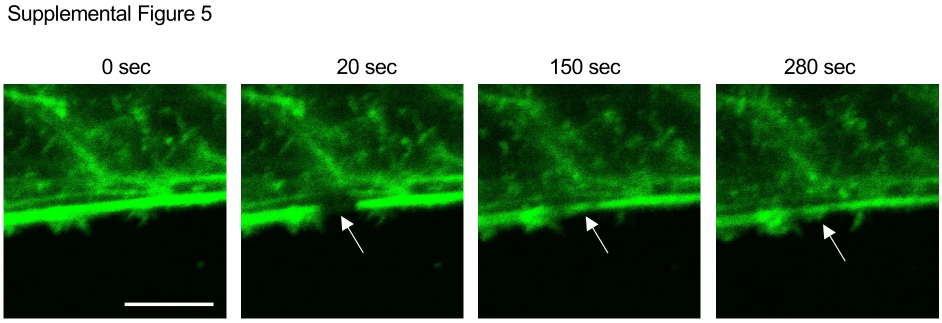

The plasma membrane of a C2C12 cell expressing actin-GFP was injured by laser. Actin was accumulated at the injured site (150 sec), which further formed a small protrusion (280 sec). Scale bar of the image is 2.5 μm.

Figure S6. Difference in cortical actin between C2C12 cells and COS7 cells.

Supplemental Figure 6

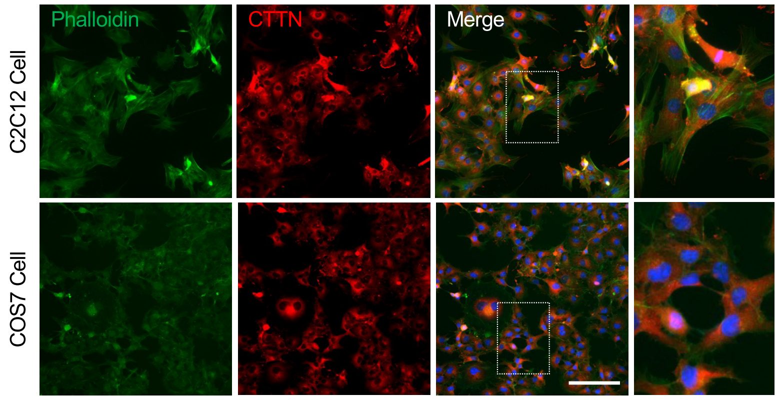

Immunostaining of CTTN (red) in C2C12 cells and COS7 cells. Nuclei and F-actin were stained with Hoechst33342 (blue) and phalloidin-FITC (green), respectively. Scale bar of the image is 125 μm.

Figure S7. Schematic summary of our hypothesis.

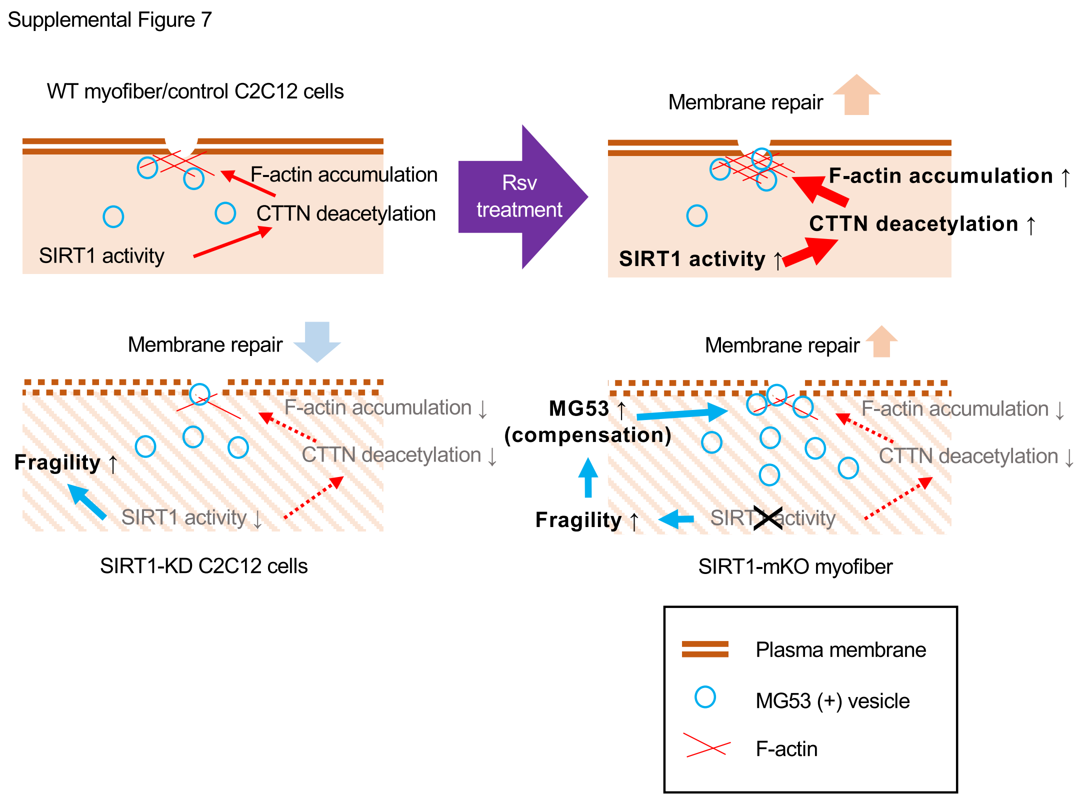

In WT mice and control C2C12 cells, Rsv treatment promotes an increase in SIRT1 activity, and upregulates membrane repair function of myofiber and cells. This concept is supported by results that suppression of SIRT1 (knockout and knockdown; KD) blocks promotion of membrane repair by SIRT1 activators in cells and mice. In SIRT1-KD C2C12 cells and SIRT1-mKO mice, supression of SIRT1 activity might reduce membrane repair through SIRT1/CTTN axis. In contrast to short-term suppression of SIRT1 in the cell model, long-term defect of SIRT1 activity in SIRT1-mKO upregulates membrane repair molecules expression, such as MG53, to compensate increased fragility of myofiber. In total, membrane repair function in SIRT1-mKO mice shows a slight improvement.
